# Primary carcinoma of the cystic duct: a case report and review of classifications

**DOI:** 10.1186/s12957-016-1073-4

**Published:** 2017-01-19

**Authors:** Lovenish Bains, Daljit Kaur, Arun Kumar Kakar, Aman Batish, Seema Rao

**Affiliations:** 1 Department of Surgery, Maulana Azad Medical College, New Delhi, India; 2 Max Super Speciality Hospital, Dehradun, India; 3 Department of Pathology, Maulana Azad Medical College, New Delhi, India

**Keywords:** Carcinoma of cystic duct, Adenocarcinoma, Common bile duct (CBD), Magnetic resonance imaging

## Abstract

**Background:**

The incidence of extrahepatic bile duct malignancies is about 2–3.6% of all gastrointestinal malignancies. Primary carcinoma of cystic duct is a rare condition comprising a fraction of all extrahepatic bile duct malignancies with less than 70 cases reported worldwide. Majority of these cases were reported from East Asia. There is paucity in such case being reported from Indian subcontinent. We present a case of primary carcinoma of the cystic duct encountered during laparoscopic cholecystectomy.

**Case presentation:**

A 65-year-old lady presented to us with symptomatic gall stone disease. Investigations revealed a distended gall bladder with multiple stones. Patient was taken up for laparoscopic cholecystectomy, during surgery a stony hard structure was found at cystic duct-common bile duct junction which was not amenable for clear dissection. Procedure was converted to open, and the patient underwent cholecystectomy with resection of common bile duct with Roux-en-Y hepaticojejunostomy and regional lymphadenectomy. Histopathological findings revealed it to be moderately differentiated adenocarcinoma of the cystic duct.

**Conclusion:**

Primary carcinoma of cystic duct is a rare condition where early diagnosis can be difficult and if accidentally detected may add to surgeon’s dilemma. Proper surgery with en-bloc resection of gallbladder, cystic duct, common bile duct, and regional lymphadenectomy is the mainstay of treatment. The prognosis of carcinoma of cystic duct is better than extrahepatic bile duct malignancies. The old classification system has outlived its time and is more rigid in definition which is not practical in advanced cases; the new classification systems of this century offer better insight into understanding the tumor characteristics and prognosis.

## Background

The incidence of extrahepatic bile duct malignancies is about 2–3.6% of all gastrointestinal malignancies. Primary carcinoma of cystic duct is a rare condition comprising a fraction of all extrahepatic bile duct malignancies with less than 70 cases reported worldwide. Majority of these cases were reported from East Asia, there is paucity in such case being reported from Indian subcontinent. It is difficult to implement strict criteria of Farrar [1] viz. (i) growth restricted to the cystic duct, (ii) absence of neoplastic process in the gall bladder, hepatic, or common bile duct, (iii) histological evidence of carcinoma; to all cases as majority of cases reported were advanced. Early diagnosis can be difficult and if accidentally detected may add to surgeon’s dilemma. Prompt surgery with en bloc resection of gallbladder, cystic duct, common bile duct, and regional lymphadenectomy is the mainstay of treatment. Adenocarcinoma is the predominant pattern; however, small cell carcinoma, carcinoid tumor, mucin-producing carcinoma also have been reported. The old classification system of Farrar [1] may not fit the current scenario due to its strict definition whereas the new classification systems [2–5] attempt to cover those shortcomings and highlight biological behaviour of this tumor.

## Case presentation

**Fig. 1:**
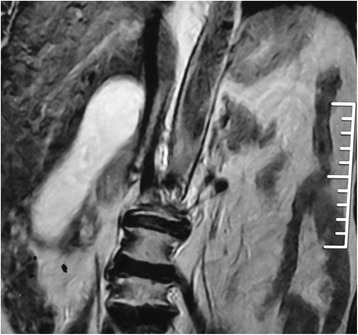
MRI showing mucocele of gall bladder (coronal plane)

**Fig. 2:**
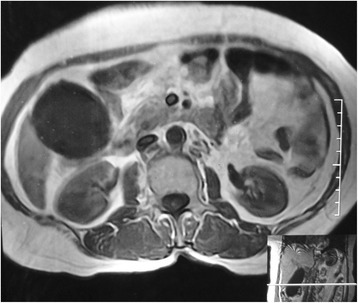
MRI showing mucocele of gall bladder (transverse plane)

A 65-year-old female presented to surgery outpatient department for recurrent right upper abdominal pain for last 3 months. There was an episode of jaundice 2 months back, which subsided spontaneously in a week. General physical examination and abdominal examination were essentially normal. Ultrasonography showed gall bladder distended (150 × 54 mm) with multiple small stones (ranging 2–5 mm), common bile duct 8 mm, no mass or lymph nodes. Investigations and laboratory parameters including liver function tests were within normal range. Magnetic resonance imaging was performed in view recent episode of jaundice which revealed mucocele of GB and no other significant findings. (Figs. 1 and 2) Patient was taken up for elective laparoscopic cholecystectomy. During surgery, a hard structure (stone/node) was found at cystic duct-common bile duct junction which was not amenable for clear dissection. The procedure was converted to open, the structure was palpated to be stony hard (adherent lymph node). No other mass or lymph nodes were noticed. The patient underwent extended cholecystectomy with resection of CBD with Roux-en-Y hepaticojejunostomy with regional lymphadenectomy. The patient made uneventful recovery and is doing well after 1.5 years of surgery.

**Fig. 3:**
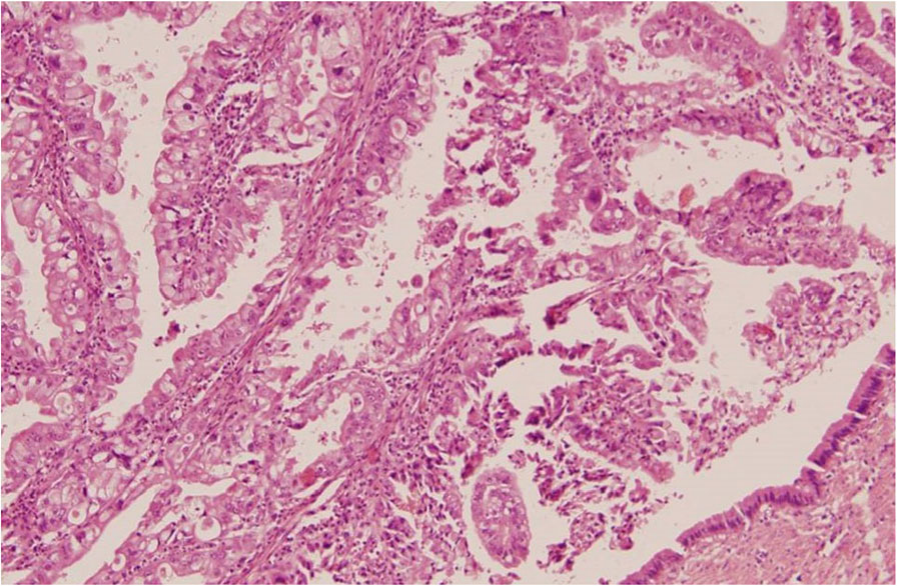
Adenocarcinoma protruding in the lumen

**Fig. 4:**
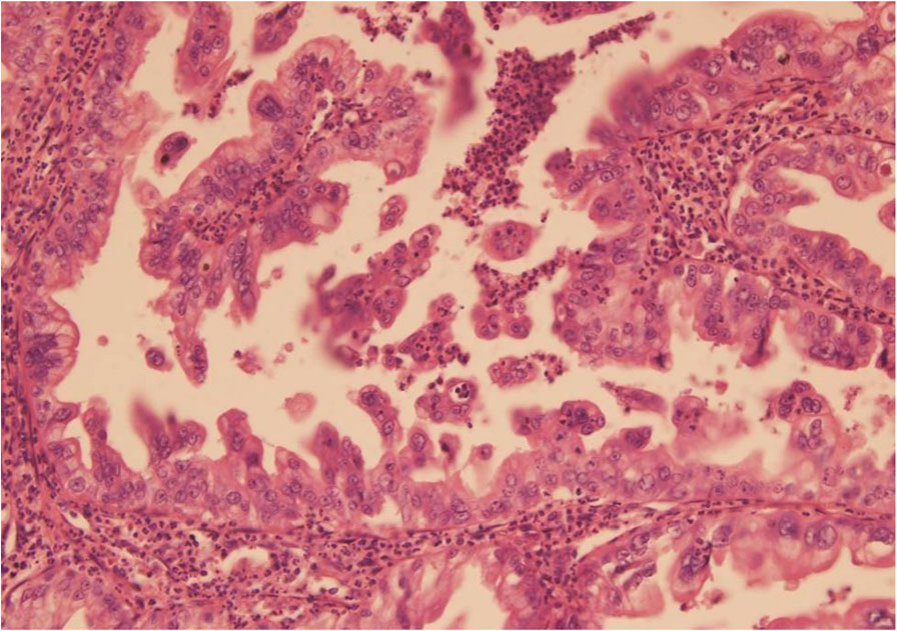
Tumor showing pleomorphism and high mitotic activity

On section cystic duct showed a polypoidal, solid growth measuring 2×1.5×1.0 cm in size and projecting into the lumen of CBD. Histological examination of the growth revealed moderately differentiated adenocarcinoma arising from duct epithelium and protruding in the lumen (Fig. 3). Tumor showed moderate pleomorphism, focal papillary pattern and high mitotic activity and was infiltrating full thickness of the duct wall and reaching up to serosa (Fig. 4). It showed perineural invasion but no lymph node metastasis. The resected margins were clear. Gall bladder was consistent with chronic cholecystitis with mucosal ulceration, focal xanthogranulomatous inflammation.

## Discussion

Primary carcinoma of the extrahepatic biliary tree has an incidence of 0.14% of all malignancies with commonest site as the CBD (40.1%) whereas primary carcinoma of cystic duct is extremely rare [5, 6]. The incidence of primary carcinoma of cystic duct in autopsy studies was found to be 0.03–0.05% [7]. The primary carcinoma of cystic duct has been found to have male preponderance with no specific sex predilection and the average age of presentation was 65 years (range, 38–79 years) [2, 4–6, 8, 9] Gallstones are not associated with all cases, being found in about 25% of the cases [2, 5, 10], and 48 and 37% in two other series [2, 9] which is in stark contrast to 75–90% reported for classical gall bladder carcinoma. It is proved that inflammation of the biliary duct epithelium due to irritation from reflux and stasis of pancreatic juice and bile leads to malignancy over a period of time [2, 7, 10, 11]. However, the risk factors for carcinoma of cystic duct are unclear.

Its clinical presentation is non-specific and mostly is similar to biliary calculus disease [2, 6, 7, 11]. The symptoms may develop earlier than GB cancer. The main symptom was abdominal pain and jaundice, both being equally distributed [1, 2, 4, 5, 8] According to Baraka et al. in 33 reported cases 81% presented with right upper quadrant abdominal pain, 41% with abdominal mass, and four cases with obstructive jaundice [6]. Gallbladder was found to be hydropic in 86 and 93% cases [2, 5]. The tumour is either discovered at laparotomy or on histopathological examination of the specimen [1, 2, 4–6, 11]. In none of the cases reported, a preoperative diagnosis was made. In our case too, patient had history of transient jaundice with upper abdominal pain and hydrops of gallbladder and final diagnosis was made on histopathological examination. Neoplasm of the cystic duct can be suspected in patients presenting with distended gallbladder likely due to cystic duct obstruction without evidence of stone impaction in the cystic duct.

Different staging systems for various cancer have been described worldwide but to date no diagnostic or staging system have been established for carcinoma of cystic duct which could be uniform and applicable to all cases [1–5]. In 1951, Farrar’s first described diagnostic criteria for primary carcinoma of cystic duct [1] (Table 1).

**Table 1:** Overview of various classifications [1–5]

	Classification	Current status
Farrar [1] (1951)	(i) Growth restricted to the cystic duct (ii) Absence of neoplastic process in the GB, hepatic, or CBD (iii) Histological evidence of carcinoma	-1st classification system but unsuitable in current scenario where advanced cases are being detected invading surrounding structures. -No mention about lymph node metastasis
Ozden et al. [2] (2003)	*working definition* of carcinoma of cystic duct is a GB tumor whose centre is located in the cystic duct (the geometric centre of the tumor)	-Practical to determine geometric centre during grossing however still may be difficult to label as cystic duct carcinoma if it had been unequal growth in different directions. -It was based on assumption that the tumor grows equally in all directions, which may not hold true for all cases.
Kim et al. [3] (2007)	(i) Type I-carcinoma confined within the cystic duct (ii) Type II-carcinoma extended to the GB neck and infundibulum or bile duct of cystic duct side without obstructive jaundice (iii) Type III-carcinoma extended up to the GB body or bile duct on the contralateral side of cystic duct opening which then causes obstructive jaundice [centre located in the cystic duct]	-Based on the extent of tumor infiltration and such classification defines treatment plan and improves resectability.
Yokoyama et al. [4] (2008)	A gallbladder tumor with centre of which is located in the cystic duct: (i) hepatic hilum type (HH)-tumor mainly invades the hepatic hilum (ii) cystic confluence type (CC)-tumor mainly involves the confluence of the cystic duct	-HH type presentation, behaviour and prognosis takes on the picture of gallbladder carcinoma, whereas CC type takes on the picture of bile duct carcinoma. -This classification may be helpful for making a diagnosis and planning the surgical procedure for individual cystic duct carcinoma patients.
Nakata et al. [5] (2009)	Based on extent of spread: Type I-the tumor was located wholly within the cystic duct Type II-the tumor extended to the gallbladder Type III-the tumor extended to the common hepatic duct or the common bile duct (including extension into the lumen and external invasion to the bile duct wall) Type IV-the tumor extended to both the gallbladder and the bile duct	-A high frequency of perineural infiltration and a low frequency of hepatic infiltration result in cystic duct carcinoma being a distinct entity from gallbladder carcinoma and better prognosis than gallbladder cancer and extra hepatic bile duct cancer.

Farrar criteria is strict and cannot differentiate advanced tumors as patients may present in different stages of the disease and *true* cystic duct carcinoma becomes a *non-cystic duct carcinoma* once it advanced beyond cystic duct [1, 2]. The new classification(s) [3–5] that defines carcinoma of cystic duct as a tumor with its center located in the cystic duct appears more practical as mostly reported cases of carcinoma of cystic duct were advanced. The cystic duct is a short structure, which lacks a proper muscle layer and consists of a thin fibro-muscular layer and adventitia. Extra hepatic bile ducts are lined by a layer of tall columnar epithelium which extends from the mucosal lining through the entire wall to form glands in the outer coats of the ducts. Thin-walled ducts, the presence of glands in the outer coats, the rich lymphatic network and nerve supply apparently facilitate spread of the tumor to periductal structures [12]. The histology of the cystic duct comprises a transient pattern from gallbladder to bile duct, indicating that there is a *watershed* in the cystic duct that separates the features of the gallbladder from those of the bile duct [4].

The classically described carcinoma of cystic duct is very rare and is attributed to its strict definition [1] in which a tumor is restricted to the cystic duct, and if new definitions [3–5] are properly applied then more cases can come up. Most of the tumors were microscopically well-differentiated adenocarcinoma, but small cell carcinoma, carcinoid tumor, mucin-producing carcinoma of cystic duct also have been reported [3, 9, 13]. Bile duct invasion is relatively rare [1, 11, 14, 15]. The incidence of lymph node metastasis is (0–40%) in patients with carcinoma of cystic duct which is lower than those with extrahepatic bile duct (about 50%) or GB cancer (40–80%) [2, 4, 11, 15].

The early development of symptoms due to obstruction of the cystic duct, mimicking signs and symptoms of gallbladder disease, slow growth, and late metastasis favor a better prognosis in patients with carcinoma of cystic duct as compared to extrahepatic bile duct or GB cancer [2–4, 6, 9, 11, 14, 15]. The perineural invasion is one of the most significant prognostic risk factor in malignancies of biliary tree. The incidence of perineural invasion in extrahepatic bile duct carcinoma is 85–93% and in gallbladder carcinoma is 24–72% [16]. High frequencies of perineural (87%) and lymphatic invasion (83.8%) were observed by Ozden et al., and similar findings were reported by Nakata et al. as perineural (73.3%) and lymphatic invasion (80%) [2, 5]. Perineural and the microscopic vascular invasion were more frequent in the HH type than in the CC type whereas lymph node metastasis and lymphatic permeation was similar in both [4].

In our case, the tumor was type 1 of Kim’s classification (confined within the cystic duct), CC type of Yokohama’s classification (in cystic duct and towards confluence of the cystic duct) and type I of Nakata’s classification (located wholly within the cystic duct). A meta-analysis conducted by Kim et al. of the previous case reports regarding clinical and pathological characteristics and patient survival found that patients with type I cancer had significantly longer survival compared to type II or III cancer (*p* = 0.018, *p* = 0.03). However, there was no significant difference between patients with type II cancer and type III cancer (*p* = 0.989) [3]. The survival rate tends to be higher for patients with the CC type than for those with the HH type (*p* = 0.064). The CC type lesions were more common in males, and female sex was predominant for the HH type [4]. It is concluded that carcinomas of cystic duct extending beyond the cystic duct are more aggressive and associated with a poorer prognosis [4, 15].

The recommended treatment is radical surgery comprising of cholecystectomy with non-anatomical gallbladder fossa resection and excision of extrahepatic bile duct with regional lymphadenectomy.^3,5,9,10.11^ The average tumor size was 25–27 mm with range of 4–60 mm [2, 5]. Postoperative adjuvant radiation therapy can be considered in cases of advanced carcinoma of cystic duct particularly those with positive surgical margin [3, 9].

The average survival was reported to be 27.2 months while that of gallbladder carcinoma was only 5.8 months and of other extrahepatic biliary ducts 3.2-11.4 months [3, 15]. Nakata et al. reported 5-year survival rate of 40% and median survival of 2.4 years. The survival was significantly longer in patients with type I as compared to type IV (*p=*0.05) whereas no significant differences in survival rate among patients with types II, III, and IV cystic duct carcinoma were found [5]. The median overall survivals for patients with HH type and CC type were 11.9 and 45.8 months, respectively [3]. Advances in imaging and molecular basis of disease have led to better understanding of this tumor however more number of cases evaluation will provide significance.

## Conclusion

This is a rare case of primary carcinoma of cystic duct encountered during laparoscopic cholecystectomy in a 65-year-old lady. The patient underwent en-bloc resection of gallbladder, cystic duct, common bile duct, and regional lymphadenectomy which remains the surgical standard of treatment. Cystic duct carcinoma may be overlooked in a patient with gallstones and hydropic gallbladder. Primary carcinoma of cystic duct, albeit rarely can be suspected in patients with distended gall bladder where imaging studies have not clearly defined a calculus. The prognosis of primary carcinoma of cystic duct is better than other extrahepatic bile duct malignancies. The old classification system has outlived its time and is more rigid in definition which is not practical in advanced cases; the new classification systems of this century offer better insight into understanding the tumor characteristics and prognosis. Further cases identified by new systems may help in proper scientific interpretation of the behavior of cystic duct carcinoma as a separate and distinct identity and standardization of classification.

## Abbreviations

CBD: Common bile duct
GB: Gall bladder
